# New Insights in Computational Methods for Pharmacovigilance: *E-Synthesis*, a Bayesian Framework for Causal Assessment

**DOI:** 10.3390/ijerph16122221

**Published:** 2019-06-24

**Authors:** Francesco De Pretis, Barbara Osimani

**Affiliations:** 1 Department of Biomedical Sciences and Public Health, Marche Polytechnic University, 60126 Ancona, Italy; b.osimani@univpm.it; 2 Department of Communication and Economics, University of Modena and Reggio Emilia, 42121 Reggio Emilia, Italy; 3 Munich Center for Mathematical Philosophy, Ludwig-Maximilians-Universität München, 80539 München, Germany

**Keywords:** advisory committees, Bayesian epistemology, data fusion, data mining, dose-responsiveness, drug approval process, *E-Synthesis*, evidence synthesis, pharmacovigilance

## Abstract

Today’s surge of big data coming from multiple sources is raising the stakes that pharmacovigilance has to win, making evidence synthesis a more and more robust approach in the field. In this scenario, many scholars believe that new computational methods derived from data mining will effectively enhance the detection of early warning signals for adverse drug reactions, solving the gauntlets that post-marketing surveillance requires. This article highlights the need for a philosophical approach in order to fully realize a pharmacovigilance 2.0 revolution. A state of the art on evidence synthesis is presented, followed by the illustration of *E-Synthesis*, a Bayesian framework for causal assessment. Computational results regarding dose-response evidence are shown at the end of this article.

## 1. Introduction

The problem of collecting, analyzing and evaluating evidence on pharmaceutical safety is a central problem of health-care practice. Statistics on drug-induced hospitalizations range between 5% and 10% of total hospitalizations in Europe as well as in all Western countries [1,2,3,4,5,6]. According to the European Medicines Agency some 197,000 people in the European Union die each year as a result of adverse drug reactions (ADRs) [7]. Drug-induced toxicities in the US, which rank among the top 10 causes of death, result in health care costs of over US$30 billion annually [8,9]. The authors of [10] estimated approximately £500 million in costs per annum for the UK as a result of adverse drug events. The annual direct costs for Germany have been estimated to amount to €400 million [11]. ADRs constitute a concern for the industry as well in that the attrition rate (the proportion of would-be drugs whose development is interrupted before reaching the market over the total research and development portfolio) is continuously increasing [12]. This concern is also one of the reasons for the development of the Innovative Medicines Initiative (IMI and IMI2) within the EU 7th and H2020 Framework Programmes [13,14]. ADRs are thus responsible for a heavy economic and social burden [15]; in addition, they constitute an extremely vulnerable point for the health system and a key ethical problem for decisions concerning pharmaceutical products.

In this landscape, evidence synthesis is now rising as a robust approach, especially in the field of post-marketing drug safety. This trend stems mainly from a public interest towards the integration of information coming from different sources when evaluating safety signals (European Parliament and the European Council: Directive 2010/84/EU; Regulation (EU) No 1235/2010; see also the 21st Century Cures Act, recently enacted in the US). An analogous wish has also been expressed by researchers in the field [16,17], and, more generally, for a broader notion and use of evidence in the medical science [18]. Despite these pressures, standard practices of evidence assessment are still based on statistical standards which are at odds with the integration of heterogeneous data [19]. Thus, there is an increasing awareness for the need of tools which support the evaluation of putative causal links between drugs and adverse reactions grounded on such heterogeneous evidence.

This article presents a new theoretical framework to support decisions in pharmacovigilance, defined “*E-Synthesis*”, a tool encoded through a Bayesian network able to robustly screen and amalgamate evidence of different types (such as epidemiological studies, molecular biology research findings, case reports) that are combined together in order to form the “critical mass” needed to make decisions about the approval/withdrawal of a particular drug for/from the market [20,21].

The paper is structured as follows: In the first part, we present an overview of the main risk detection methods currently available to decision makers in pharmacosurveillance. In the second part, we present the theoretical underpinnings and the epistemic working of *E-Synthesis*: An instrument that uses Bayesian nets in order to exploit the joint evidential force coming from different lines of evidence. In the third part, we focus on one such line of evidence, namely the support provided by dose-responsiveness (*DR*). We draw on computational methods currently employed to process dose-response evidence in pre-approval trials [22,23,24,25,26,27] and embed them in our theoretical framework; a final discussion follows.

## 2. State of the Art

A series of directions have been explored in the field of collecting, evaluating and summarizing evidence of harm in pharmacosurveillance. Tools are subdivided according to: (i) The kind of evidence they allow to be taken into account in risk detection/assessment; (ii) and types of methods used to assess and measure it.

### 2.1. Aggregation of Spontaneous Reports

Spontaneous reports are a fundamental source for post-marketing drug safety, a set of information that is particularly valuable today when coupled with the current computational methods employed for big data analysis. FAERS (Food and Drug Administration (FDA) Adverse Event Reporting System) (https://www.fda.gov/Drugs/GuidanceComplianceRegulatoryInformation/Surveillance/AdverseDrugEffects/) implemented in the US, and EUDRAVIGILANCE (European Union Drug Regulating Authorities Pharmacovigilance) in the EU largely embodies this vision. (https://www.ema.europa.eu/en/human-regulatory/research-development/pharmacovigilance/eudravigilance. Differently from FAERS, EUDRAVIGILANCE tackles not only postmarketing safety surveillance but is aimed at reporting and evaluation of suspected adverse reactions since the very development of new drugs).

At a global scale, a well-known reservoir of data in this field is VigiBase (https://www.who-umc.org/vigibase/vigibase/), a unique WHO international database of individual case safety reports—the largest of its kind in the world—with over 19 million reports of suspected adverse effects of medicines, submitted since 1968 by member countries of the World Health Organization (WHO) Programme for International Drug Monitoring. VigiBase is continuously updated with incoming reports. A special focus has been put on spontaneous reports processing and a series of analysis algorithms aimed at the discovery of potential pharmacological harms has been created [28]. The most significant ones are: vigiRank, vigiGrade, and vigiMatch.

vigiRank [29] is a predictive model implemented in signal detection that ranks pharmacovigilance safety signals according to multiple aspects of strength of evidence. Parameters that make up the score are: Disproportionate reporting, recent reporting, geographic spread, informative reports and narratives included in case reports.

vigiGrade [30] is a multidimensional measure of the amount of information on reports that highlights quality issues in collection of individual case safety reports and gives a score for how complete a case report is. The main use of vigiGrade is as part of communication between countries belonging to the WHO on data quality, but it has also proven to be an indicator of a true signal and is therefore one of the parameters included in the vigiRank method used by the WHO in signal detection. vigiMatch [31] is a probabilistic record matching method, a likelihood-based approach to identify unexpectedly similar record pairs in large databases. vigiMatch acts as a data cleaner and computes a match score for each pair of records, where matching information is rewarded and mismatching information penalised. This score reflects the probability that the two records relate to the same underlying entity or that they are duplicates. Record pairs with match scores that exceed a certain threshold are flagged as suspected duplicates. The threshold is derived from a comparison between the match scores of confirmed duplicates and of random record pairs in the database of interest.

### 2.2. Aggregation of Human and Animal Data

The extrapolation of animal data to estimate effects on humans has in general proved to be very fruitful, especially in fields where hazard measurement definitely results to be complex and awkward. However, animal-human extrapolation is notoriously fraught with difficulties [32]. Integration of animal-human data is a viable solution to some of the problems raised by extrapolation. Regarding chemicals, guidance in this respect has been available for a long time [33]. In particular, ECETOC ([33], chapter 7) has identified the need to review and evaluate the different types of human data that are available, and to provide guidance on how such data could be used best in the risk assessment process. The proposed framework considers when and where human data could be used to support risk assessment and risk management decisions and how human and animal findings could be integrated and used in tandem. Its related model examines the quality of human and animal data and evaluates the risk, by merging all the information through an integrating matrix.

### 2.3. Bayesian Aggregation of Safety Trial Data

Bayesian methods have been proposed to substitute a frequentist approach in evaluating and aggregating evidence, especially in designing, monitoring and reporting randomized controlled trials (RCTs). Researchers like [34] claim that a Bayesian theory of statistical inference may provide a better match to the dynamic nature of clinical investigation, a context in which knowledge accrues across multiple studies and observations and in which synthesis of evidence often provides a more definitive answer to a particular question of interest. Through a Bayesian approach, accumulating data are used to formally update prior distributions via Bayes rule, yielding posterior distributions that in turn become prior distributions awaiting further data. An implementation of Bayesian methods for designing RCTs is described by [35] where the authors propose a pragmatic approach to Bayesian analysis of randomized trials that avoids the complexity but retains the core features of more elaborate Bayesian approaches. On the same perspective, a similar outlook has been taken for the combination of treatments in clinical trials by [36]. Moreover, a nice overview of the use of Bayesian methods to aggregate evidence in designing RCTs is provided by [37]; additional excellent references include [38,39,40].

### 2.4. Data Mining and Fusion Methods

Since their emergence, data mining methods have been considered a natural realm for evidence synthesis, especially in a pharmacosurveillance context. Over the years, signal detection within data of pharmacological interest has been carried along with the progresses of computer science and the availability of increasing computational power. In such contexts, data mining techniques have been deployed as fast and efficient ways to detect possible ADR signals coming from different sources of evidence. Several data mining algorithms have been described in literature [41], mainly based on nonparametrical statistics theory, including the Reporting Odds Ratio (ROR), Multi-item Gamma Poisson Shrinker (MGPS), the Proportional Reporting Ratio (PRR), and the Information Component (IC) [42] to name a few examples. An overview of statistical signal detection methods has been outlined in [43], whereas the first seminal examination of data mining techniques was given by Hauben over several articles [44,45,46]. Though successful and broadly implemented also by the pharma-industry sector [47], the theoretical basis and limitations of these data mining methods should be understood by researchers and drug safety professionals, highlighting that such automated methods should not be mechanically accepted [48]. Furthermore, the published evaluations of these techniques are limited mainly to large regulatory studies and performance characteristics may differ from case to case, principally in the smaller security databases of drug developers; therefore, it is worthwhile to remark that conclusive comparisons of the main techniques have not yet been established and such methods still have to be considered an experimental field of research. Eventually, the mathematical basis of such techniques shall not obscure the numerous data confusion and distortion factors [44].

In partial response to such criticism, data fusion methods have been introduced. Data fusion has been defined as the process of integrating multiple data sources to produce more consistent, accurate, and useful information than that provided by any individual data source [49]. From this perspective, many research efforts in pharmacosurveillance can be seen as data fusion driven. For example, ref. [50] estimates the increased risk of drug-induced hyponatremia during high temperatures based on various kind of data such as administered drugs, onset dates, given causality assessments, sodium levels and geographical origin of the ADRs reports.

Given their versatility in processing information from multiple sources, data fusion methods are considered a promising technique for evidence synthesis. In fact, as stated by [51], classical data mining methods for drug safety signal detection have mostly been limited to the examination of one adverse event data stream at a time, whereas data fusion methods for signal detection are explicitly designed to involve multiple data streams, with the goal of detecting safety signals more quickly and reliably using multiple data streams relevant to drug safety. However, shifting to this new paradigm requires drug safety researchers to design and implement novel data fusion algorithms, mainly needed for: (i) Automated data cleansing; (ii) vocabulary mapping and control across cross-functional databases; (iii) statistical detection of safety signals using multiple data streams; (iv) incorporating clinical trial data into the signal detection problem; (v) incorporating known clinical knowledge, or context, into all aspects of the problems of pharmacosurveillance. An overview of possible solutions related to this problem is offered in [51,52].

### 2.5. Semantic (Web) Methods

Semantic techniques are emerging as another trend in evidence synthesis. Researchers in [53] state that semantic technologies may provide the means to address some of the challenges in processing evidence and synthesizing it within a pharmacological context. Particular emphasis is on: (i) Annotating data sources and analysis methods with quality attributes to facilitate their selection given the analysis scope; (ii) consistently defining study parameters such as health outcomes and drugs of interest, and providing guidance for study setup; (iii) expressing analysis outcomes in a common format enabling data sharing and systematic comparisons; (iv) assessing/supporting the novelty of the aggregated outcomes through access to reference knowledge sources related to drug safety. According to [53], a semantically-enriched framework can facilitate seamless access and use of different data sources and computational methods in an integrated fashion, bringing a new perspective for large-scale, knowledge-intensive signal detection. Furthermore, the implementation of semantic algorithms finds a natural landing on web-based data. Authors in [54] explicitly tackle this perspective, offering a pharmacovigilance model based on a semantic web-based platform for continuous and integrated monitoring of ADRs in open data sources and social media.

## 3. *E-Synthesis*: A Bayesian Epistemology-Driven Framework for Pharmacovigilance

The methods presented in the previous sections are well developed and established; however they are mainly focused on specific kinds of evidence and methods. We present here a broader, holistic approach, which intends to exploit the *joint contribution* of different kinds of evidence to the (dis)confirmation of hypotheses of causal assessment: *E-Synthesis*.

*E-Synthesis* emerges as a theoretical framework for causal assessment based on [20,21], aimed to support estimates of causal associations between drugs and adverse events. *E-Synthesis* practically consists in a “scaffold” where all the available evidence may probabilistically increase or decrease causal hypotheses. (Note that the framework does not aim to provide utilities of harms, nor probabilities of expected benefits, nor utilities of benefits. When utilities of harms and benefits, as well as estimation of benefits are provided by further means, a drug regulatory agency can perform an expected utility calculation to determine whether the expected advantages of a drug exceed the expected dis-advantages. The agency will withdraw the drug (or not approve it), if the expected disadvantages outweigh the expected benefits, see ([20], Section 2.2)).

The **hypothesis of interest** is: ‘Drug *D* causes harm *E* in population *U* and model M”. To facilitate the inference from all the available evidence, **indicators of causality** are used. These indicators are based on Hill’s nine viewpoints for causal assessment [55]. Below, we detail the main components that build up *E-Synthesis*.

### 3.1. Bayesian Network Model

**Figure 1:**
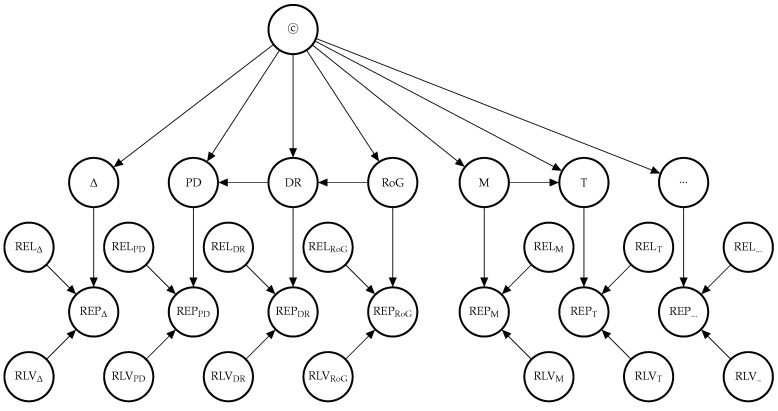
Graph of the Bayesian network with one report for every causal indicator variable. The dots indicate that there might be further indicators of causality not considered here. As explained in text, we here take it that *M* entails *T* and hence introduces an arrow from *M* to *T* which is not in [20]. *REL* and *RLV* act as evidential modulators of data (*REP* nodes).

In *E-Synthesis*, the probability of the causal hypothesis *©* is modelled via a Bayesian network linking a finite number of propositional variables, see ([20], Section 5). The ”root” variable represents the hypothesis of causal association (‘Drug *D* causes harm *E* in population *U* and model M”), its children represent abstract “causal indicators”, such as statistical association (“Probabilstic Dependence”, *PD*), dose-response (*DR*), rate of growth (*RoG*), etc. Every concrete study result probabilistically confirms one or more of such indicators with higher or lower strength, depending on its reliability (internal and external validity). The latter is gauged by “evidential modulators”, see Figure 1.

**Figure 2:**
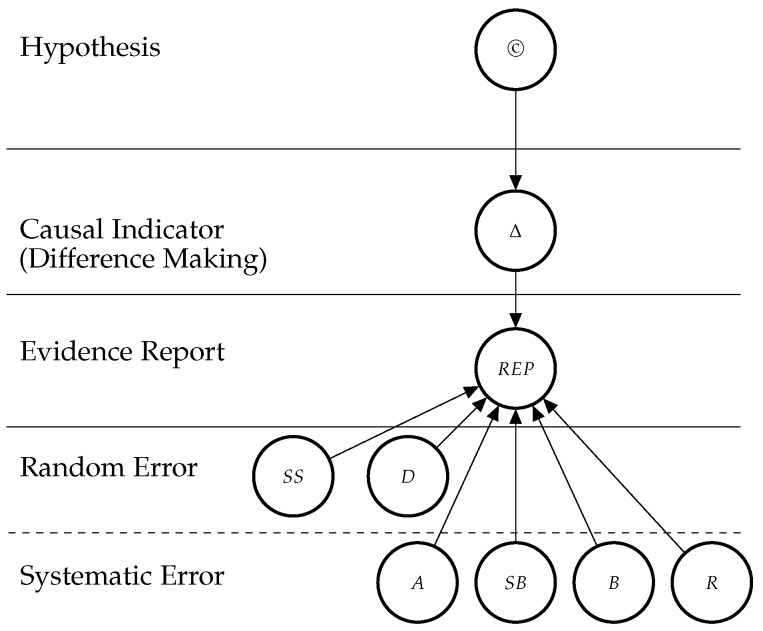
Graph structure of the Bayesian network for one randomised controlled trial (RCT) which informs us about difference making (Δ) which in turn informs us about the causal hypothesis. The information provided by the reported study is modulated by how well the particular RCT guards against random and systematic error.

The conditional probabilistic (in-)dependencies can be gleaned from the graph in terms of [56]’s *d*-separation criterion. While the Bayesian network is used for causal assessment, the arrows in the network are *not* causal arrows in [56]’s sense—here they represent epistemic probabilistic (in-)dependencies only. Figure 2 is an example graph with only one report. The causal hypothesis represented by (*©*) is a root node (The abbreviations and symbols used here are listed at the end of the manuscript). The causal indicators are parents of report nodes which mediate causal inference from the concrete data (the reports) towards the causal hypothesis. The parents at the two bottom levels are modulators of the evidential strength of the data. These incorporate considerations about the reliability of the evidence into the assessment of the hypothesis. In particular, they take into account the possibility of random error (as a function of sample size (*SS*) and study duration (*D*)), and systematic error, attenuated by adjustment or stratification (*A*), randomisation (*R*) and blinding (*B*), and favoured by sponsorship bias (*SB*).

This framework also allows for the incorporation of evidential modulators related to external validity (called “relevance” in [20]), however we will not treat them here for ease of exposition, since they are extensively addressed in [57].

A probability function consistent with the conditional independencies of the Bayesian network is selected which expresses our uncertainties in the tradition of Bayesian epistemology [58,59,60]. In case one does conditionalise on a particular model, conditional probabilities are (virtually) always set to the probabilities of the statistical model. However, unlike in “pure” Bayesian statistics, where one conditionalises on statistical models and hence obtains conditional probabilities mandated by the particular model (parameter), in Bayesian epistemology one may conditionalise on any proposition (or event), since probabilities are interpreted more widely as one’s uncertainties about general propositions; the Bayesian statistician Lindley is sympathetic to this approach [61].

### 3.2. Theoretical Entities

Concepts of interest fall into two classes: (i) A class of causal concepts comprised of the hypothesis of interest and the indicators of causation and (ii) a class of evidential concepts including evidential modulators and reports (data).

#### 3.2.1. The Causal Hypothesis (*©*)

We are interested in determining the probability of the causal hypothesis that a drug *D* causes a particular adverse effect *E* in a population *U*—given the available evidence. While the hypothesis space in principle could be subdivided into three hypotheses: (1) *D* causes *E*, (2) *D* hinders *E*, and (3) *D* does not cause *E*, we divide it here for simplicity’s sake into two alternative hypotheses: (1) *D* causes *E* and (2) *D* does not cause *E*, which consists of the disjunct of 2 and 3 above. To shorten notation, we use the symbol *©* in order to denote causation, such as in *D©E*, or simply *©*. (We do not commit here to any specific view or definition of causation (e.g., dispositional, probabilistic, counterfactual, manipulationist, etc., see also [20]. That is a question on the ontology of causation that we leave open for the moment. Our causal hypothesis allows for the term “causes” to cover any of the current definitions of causality to the extent that the evidence used for causal inference may be made relevant to them).

#### 3.2.2. Indicators of Causation

Causal inference is mediated in the framework by “indicators of causation” in line with the Bradford Hill Guidelines for causation. As Hill puts it [55]:

“None of my nine viewpoints can bring indisputable evidence for or against the cause-and-effect hypothesis and none can be required as a sine qua non. What they can do, with greater or less strength, is to help us make up our minds in the fundamental question—is there any other way of explaining the set of facts before us, is there any other equally, or more, likely than cause and effect?” (Bradford Hill both refers to explanatory power and likelihood as reliable grounds to justify causal judgements, and presents the respective criteria as opposed to tests of significance: “No formal tests of significance can answer those questions. Such tests can, and should, remind us of the effects that the play of chance can create, and they will instruct us on the likely magnitude of those effects. Beyond that, they contribute nothing to the proof of our hypothesis.” [55])

In epistemic terms, causal indicators can be considered as observable and testable consequences of causal hypotheses. They are however indeterministic consequences of such hypotheses; that is they are more likely to be observed than not in the presence of a causal relationship, but they are not entailed by it. However, the first indicator (“difference-making”, Δ) is a perfect one, in that it entails causation (although it is not entailed by it).

**Difference-making (Δ)**: If *D* and *E* stand in a difference-making relationship, then changes in *D* make a difference to *E* (while the reverse might not hold). In contrast with mere statistical measures of association, the difference-making relationship is an asymmetric one. Probabilistic dependence can go in both ways (e.g., if *Y* is probabilistically dependent on *X*, then also *X* is probabilistically dependent on *Y*); the same does not hold for difference making, which provides information about its direction. This explains why experimental evidence is considered particularly informative with respect to causation; the reason is exactly that in experiments, putative causes are intervened upon, in view of establishing whether they make a difference to the effect. (In philosophical terms, difference-making is understood as *ideal controlled variance* along the concept of *intervention* in manipulationist theories of causation (see [20] for a detailed treatment, see also [62] and [56]): *X* is called a cause of *Y* if *Y*’s value can be varied by varying *X* (possibly upon controlling for additional variables in the given situation)). Hence, randomised controlled trials (RCTs) are the privileged source of evidence for Difference-making (Δ): Reports from RCTs contribute to the (dis)confirmation of causal hypotheses via the Δ node.

**Probabilistic dependence (*PD*)**: *PD* encodes whether *D* and *E* are probabilistically dependent or not—such dependence naturally increases our belief in some underlying causal connection (as an indicator of causation; see, e.g., [63]). Probabilistic dependence is an imperfect indicator of causation because neither the former entails the latter nor the reverse. There are cases in which probabilistic dependence is created by confounding factors, and cases where two opposite effects of a single cause cancel each other out and produce a zero net effect. (A well-known example of this type of cancellation is Hesslow’s birth control pills case (see, e.g., [64]): The contraceptive (directly) causes thrombosis but simultaneously (indirectly) prevents thrombosis by preventing pregnancy which is a cause of thrombosis. Cartwright ([64]) discusses this case as one of the pitfalls of reducing causal analysis to probabilistic methodology alone. Of course, if cancellation is suspected, one might disable certain preventative causal routes to check whether the causal relationship actually shows once disabling conditions are held fixed. Cartwright however discusses cases in which this strategy might not even be viable, owing to the complexity of the causal web.).

**Figure 3:**
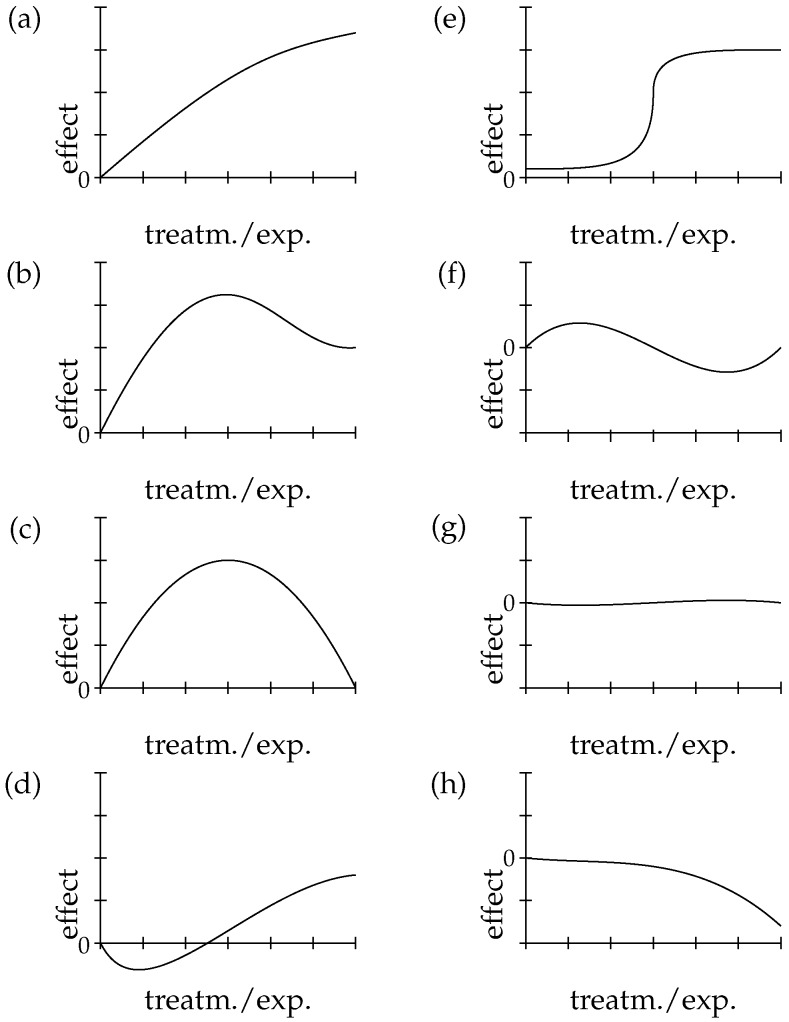
Possible functional forms of the relationship between *D* and *E*. Adapted from [22] (Figure 1).

**Dose-response relationship (*DR*)**: Dose-response relationships are taken as strong indicators of causation. *DR* is a stronger indicator than probabilistic dependency alone, because it requires the presence of a clear pattern of ≥3 data-points relating input and output. Indeed *DR* implies *PD*. Dose-response relationships can be inferred both at the population and at the individual level, and both in observational and experimental studies. *DR* abstracts away from these specifications and means that for dosages *D* > 0 in the therapeutic range, the adverse effect *E* shows (approximate) monotonic growth for a significant portion of the range (see below, Figure 3, for an illustration of important types of dose-response curves).

**Rate of growth (*RoG*)**: This indicator signals that the dose-response relationship is a steep one. If we have evidence of *DR* and evidence of $\bar{RoG}$ means either that the rate of growth is low, or highly non-linear.

The indicators of causality *RoG*, *DR* and *PD* are independent of the causal structure, in the sense that they could be equally observed either in cases where *D* causes *E*, or in cases where *E* causes *D*, or when *D* and *E* have a common cause. All that matters is whether there is a (certain) systematic relationship between *D* and *E*. *RoG*, *DR*, *PD* are semantically and epistemically related and we refer to them as “statistical black-box indicators”, denoted by Σ.

**Mechanistic Knowledge (*M*)**: *M* represents the proposition: “there is a mechanism”, meaning that there is a physiological pathway from drug use to the effect. In the biological realm, a causal relationship obviously entails the presence of a biological pathway connecting the cause to the effect. Therefore, © ⇒ *M*. However, this pathway may not be causally responsible for bringing out the effect due to possible inhibitors, back-up mechanisms, feedback loops, etc. *M* ⇒ © does hence not necessarily hold.

**Time course (*T*)**: *T* encodes whether *D* and *E* stand in the right temporal relationship (time course), which can refer to temporal order, distance, or duration. If *D* causes *E*, *T* must hold (as a necessary condition): © ⇒ *T*. *T* remains an imperfect indicator, nevertheless, because temporal precedence is also compatible with ¬(*D* causing *E*) while *D* and *E* are connected by a common cause or through reversed causation. Hence *T* ⇒ © does not necessarily hold.

According to our view, study design determines the kind of information that the evidence is able to provide us, hence in the following we define the evidence as a function of the kind of information it delivers: That is, the causal indicator it “speaks” to.

We associate study designs with causal indicators as follows: RCTs provide information about difference making, time course, possibly also dose-response relationship and rate of growth. Cohort studies provide evidence of time course and statistical association (Σ). Case-control studies provide information about Σ only. Individual case reports cannot provide information about statistical association, but they provide very detailed information about time course and possibly difference-making whenever this can be established with confidence (see for instance the Karch-Lasagna or Naranjo algorithm [65,66,67]). However, such information is “local”, that is about an individual subject, and therefore do not license inferences about the general population. A case series can then possibly help delineate a reference class, where the putative causal link holds. Basic science studies (in vitro, or in silico), and in vivo studies, are generally the main source for evidence on the mechanisms underpinning the putative causal link.

## 4. Zooming in *E-Synthesis*: Processing Evidence for Dose-Response

As outlined above, in *E-Synthesis* many indicators of causality contribute to the assessment of pharmacological risk. Among them, we decide in this part to focus on Σ, and, in particular, on the *DR* arm. This choice is motivated by the fact that dose-responsiveness has been investigated for a long time as a manifest signal of pharmaceutical harm in pharmacosurveillance [68] (non-dose-related ADRs have been known and studied in the field too [69]).

In [21] we presented the epistemic dynamics of *E-Synthesis* by applying it to a case study: The debated causal association between paracetamol and asthma. We focus here on the *DR* arm. [70] is a study supporting the claim of paracetamol and asthma association, based on evidence pertaining to both *DR* and *T*. The study reports a prospective cohort study for women in which, between 1990 and 1996, 73,321 subjects were included in the analysis. Proportional hazard models included age, race, socioeconomic status, body mass index, smoking, other analgesic use, and postmenopausal hormone use. During 297,282 person-years of follow-up, 299 participants reported a new physician diagnosis of asthma meeting diagnostic criteria (see Table 1). For this study, in [21] we derived:

(1) $$P(ES=1|\vec{x},DR,T)=1$$

and:

(2) $$P(ES=1|\vec{x},\bar{DR},\bar{T})=0$$

where *ES* stands for the observed effect size (with significant = 1, not significant = 0) and $\vec{x}$ is a vector containing all the modulators introduced in the previous part. Evaluating such research study allows one to quantify the probability of witnessing a proxy of hypothesis of causation (i.e., the effect size, in this case) conditioned on the presence of dose-responsiveness and temporal precedence. This example highlights well that *E-Synthesis* is able to process the given evidence, but this process occurs through the mediating “lenses” of an evaluation of research studies, meaning that all the available evidence has always been analyzed ab initio by other researchers.

Nevertheless, from a more general point of view, a preferred approach would be to compute the above probabilities directly, by treating the evidence at glance. If we focus on the *DR* arm, what we would like to compute is thus:

(3) $$P(DR|E_{DR},\vec{x})$$

that is the probability of dose-responsiveness, given the available dose-response evidence and its related modulators (From this point onward, our problem will be approximated by the study of $P(DR|E_{DR})$. The introduction of modulators into this framework will be addressed in a forthcoming article.)

Hence, a key question here is which may be the most suitable computational methods to be implemented to process *DR* evidence with the final goal of making a sound estimate of such probability. Regarding this problem, in recent years an extensive literature has been developed not directly in the realm of pharmacosurveillance but more specifically for dose-finding assessment, a task that is usually targeted in Phase II of clinical research. In [22], an algorithm aimed at this task named Multiple Comparison Procedures and Modeling (MCP-Mod) is presented. This algorithm is composed of two main steps. The ”MCP” step which includes: (i) The assessment of a dose-response signal using a suitable test for trend; (ii) the model selection (or model averaging) out of the set of statistically significant dose-response models. The ”Mod” step focuses on dose-response and target dose-estimation based on the previous selected model.

Given our interest in pharmacovigilance, we will focus only on the “MCP” step. The mentioned test for trend is a data analysis procedure able to demonstrate a dose-response association between the risk factor (drug prescription) and the outcome (adverse event), even if the association is not statistically significant for any particular level of exposure. A clear description of this test (also known as p-trend test or just p-trend) is presented in [71], whereas a good mathematical exposition can be found in [72]. A discussion regarding the use of confidence intervals instead of *p*-values in such test is analyzed in [73].

The model selection represents the second part of the “MCP” algorithm. Here, given some kind of association having been established in the test for trend analysis, the focus is on the detection of the best dose-response pattern. In other words, if we look back at Figure 3, our goal here is to select which kind of panel best fits the results obtained by the p-trend test. For instance, if we consider the (g) panel, the final outcome is non dose-responsiveness, whereas the (e) panel presents a sigmoid *DR* pattern. As shown in ([22] Table 5), every model we may select has an associated probability computed on the given evidence. However, our main interest is limited to discriminating between a horizontal pattern (i.e., signalling $\bar{DR}$) and a non horizontal one, whatever functional form it may exhibit. This may offer a welcome shortcut to the computation of $P(DR|E_{DR})$.

Since its introduction in [22], the MCP-Mod algorithm has been subject of subsequent research, with various enhancements presented in [23,24], with highlights also on subgroups analysis [25] and extensions to a continuous set of possible dose-response patterns [26]. A Bayesian approach for dose-finding has been also proposed in several papers [74,75,76,77]. In particular, in [27], we find an explicit formula for calculating *P*(*data*|*θ*) where *θ* represents a set of parameters embodied in a statistical model *f*(*d_i_*|*θ*) that defines the possible functional patterns of dose-responsiveness exhibited in Figure 3.

(4) ln[P(data|θ)]=∑i=1Glnniyi+yiln[f(di|θ)]+(ni−yi)ln[1−f(di|θ)]

In this formula, *d_i_* represents the dose-level, *n_i_* the number of subjects in each dose-group, *y_i_* the number of subjects with effect in the corresponding dose-group and *r_i_* is the dose-response computed as the ratio *y_i_*/*n_i_*. The index *i* is a natural number less or equal to *G*, the number of dose-groups.

We exploit Equation (4) to compute $P(data|\bar{DR})$. In fact, beyond many statistical models representing dose-responsiveness, a non dose-response pattern is expressed only by a family of constant functions, that is *f*(*d_i_*|*θ*) = *k* where *k* is a real number subjected to the following bound: 0 ≤ *k* ≤ 1. Among all the possible values of *k*, it is possible to calculate the optimal one ($\tilde{k}$) through a least squares regression analysis based on the given dose-response (*r*) data. That can be achieved by solving this problem:

(5) $$\tilde{k}=\underset{k}{\arg\min}\sum_{i=1}^{G}{\left(r_{i}-k\right)}^{2}=\langle r\rangle$$

The bracket-operator 〈·〉 expressed in Equation (5) coincides with the arithmetic mean over the set R containing the given dose-response (*r*) data. (Instead of using a common overline notation to express the arithmetic mean (i.e., $\bar{r}$), we prefer to introduce here the bracket-operator 〈·〉, since the overline already appears for the nonoccurrence of events in the probabilistic formulas.).

Given this information, we can now compute the likelihood of the data, given the absence of any dose-response pattern $\bar{DR}$:

(6) ln[P(data|DR¯)]=∑i=1Glnniyi+yiln〈r〉+(ni−yi)ln(1−〈r〉)

Equation (6) turns out to be very useful to compute $P(DR|E_{DR},\vec{x})$. In fact, through the Bayes theorem is easy to prove that:

(7) $$P\left(DR|data\right)=1-P\left(\bar{DR}|data\right)=1-\frac{P\left(data|\bar{DR}\right)P\left(\bar{DR}\right)}{P(data)}=1-\frac{P\left(data|\bar{DR}\right)\left[1-P\left(DR\right)\right]}{P(data)}$$

where P(data) can be written as:

(8) $$P\left(data\right)=P\left(data|DR\right)P\left(DR\right)+P\left(data|\bar{DR}\right)P\left(\bar{DR}\right)$$

In order to compute such expression, it appears crucial to determine which statistical model *f*(*d_i_*|*θ*), i.e., which functional pattern of dose-responsiveness has to be used in Equation (4). Here, we do not commit to any particular kind of likelihood but we point at the fact that only data-analysis can provide a correct estimation for Equation (8). Moreover, Equation (7) requires the estimation of *P*(*DR*), that is the a priori probability of dose-responsiveness. This probability can be estimated through clinical considerations regarding the expectation of dose-responsiveness for a certain drug, or, more generally, in a specific field of scientific inquiry. The degree of specificity of such a prior depends on the available knowledge.

We now present a numerical example of this new methodology to assess $P(DR|E_{DR},\vec{x})$. We again consider the study [70]. By analyzing the data shown in Table 1 (In order to make a more unbiased analysis, data have been divided into two separate sets, one used for model estimation and the other one for the computation of probabilities.), we compute different likelihood and evaluate their performance according to several established criteria for curve fitting, such as the Akaike information criterion (AIC), the Bayesian information criterion (BIC), *R*^2^ and its adjusted version. Such analysis is outlined in Table 2: The exponential model emerges as the best according to all criteria (AIC, BIC, *R*^2^ and adjusted *R*^2^), whereas the linear and the cubic models perform worst.

From the data, it is possible to estimate the likelihood for non dose-responsiveness:

(9) $$\ln[P\left(data|\bar{DR}\right)]\approx -25.903$$

(10) $$P\left(data|\bar{DR}\right)\approx 5.63\times 10^{-12}$$

and, for instance, the related likelihood for *DR* according to an exponential model: Table 3 presents the predicted values that are neeeded for such computation.

(11) $$\ln[P\left(data|DR_{exp}\right)]\approx -18.6327$$

(12) $$P\left(data|DR_{exp}\right)\approx 8.09\times 10^{-9}$$

If we again consider Equation (7), we conclude that:

(13) P(DR|data)≈1

**Figure 4:**
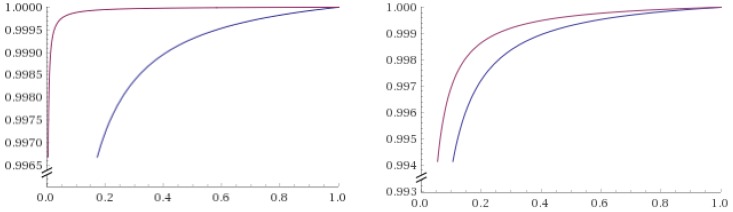
This figure shows the conditional probability *P*(*DR*|*data*) computed for the data presented in Table 1. X-axis represents *P*(*DR*) and Y-axis *P*(*DR*|*data*). On the left panel, *P*(*DR_exp_*|*data*) (blue line), embodying an exponential likelihood for *DR*, is pictured against *P*(*DR_cub_*|*data*) (violet line), calculated by using a cubic likelihood. On the right panel, *P*(*DR_exp_*|*data*) (blue line) is again pictured against *P*(*DR_lin_*|*data*) (violet line), computed with a linear likelihood.

This results are consistent with what was previously done in [21] and shown in Equation (1). In Figure 4, we present the curves associated to various *P*(*DR*|*data*) for the best and worst tested statistical models at varying levels of *P*(*DR*).

## 5. Discussion

Adverse drug reactions present a significant problem to drug manufacturers, are a serious risk to patients, and constitute a major ethical problem in licensing decisions of pharmaceutical products. Current standards for drug evaluation are based on a methodology developed for the assessment of benefits that focuses on internal validity and privileges randomized controlled trials. However, evidence for harm often emerges unsystematically and unpredictably in form of anecdotal reports, case series and survey data. Hence, this methodology faces overwhelming challenges in integrating such diverse evidence.

Whereas a sophisticated set of tools for meta-analysis and systematic reviews has been developed for the purpose of evaluating intended effects of interventions, the adaptation of these instruments with the aim of evaluating safety of health technologies encounters various problems, mainly due to the (i) relative scarcity, (ii) heterogeneity, and (iii) “fragility” of data concerning unintended effects of medical treatments: (i) Evidence about unknown risks emerges gradually from spontaneous reports or other kinds of sporadic data: Especially in the earlier phases of risk detection, these data can be at the same time very noisy and rare; (ii) furthermore, they may come from heterogeneous sources (such as clinical case series, animal studies, or molecular studies, etc.); (iii) and it can be unreliable, because of noise and bias. Risk minimization requires however, that timely decisions are made on the basis of the available, albeit inconclusive and possibly “low-quality” evidence.

One major obstacle for the optimization of the kind of diverse evidence that we encounter in pharmacosurveillance is inherited from the epistemology underpinning the standard methodological paradigm entrenched in the medical community, i.e., frequentist hypothesis testing. This only works with predefined research protocols and can pool only homogeneous data. Thus, current approaches lack the justificatory underpinning for anecdotal reports, case series, or survey data, which are the main source of evidence for drug safety assessment. Such a perspective, together with the implementation of new computational methods [78], challenges the structure of decision making processes within the pharma-industry and the regulatory bodies.

Starting from these premises, in Section 2 we have shown ongoing trends in evidence synthesis methods. There, we have presented a state of the art of current methods employed to assess and measure risk in pharmacovigilance, highlighting in particular aggregation of spontaneous reports and data mining and fusion techniques.

Following this path, in Section 3 we have outlined *E-Synthesis*, a Bayesian framework for causal assessment that represents a new approach to evidence synthesis grounded on theoretical bases [20,21]. *E-Synthesis* can be pictured as a Bayesian network able to synthesize evidence of different types (such as epidemiological studies, molecular biology research findings, case reports) that are combined together in order to form the “critical mass” needed to make decisions about the approval/withdrawal of a particular drug on the market. Through several indicators of causality (difference-making, probabilistic dependence, dose-response relationship, rate of growth, mechanistic knowledge and time course), *E-Synthesis* acts as a theoretical *ordering scaffold* on the available evidence and arrange it in order to assess the causal relationship between a certain drug and harm in a given population.

Among the indicators of causality, we have focused here on dose-responsiveness (Section 4). In particular, we have drawn on current methods applied in clinical research for dose-finding and tried to embed them in *E-Synthesis*. By combining different approaches and frameworks (MCP-Mod [22], Bayesian benchmark dose-estimation [27]), we have provided a new model to estimate the probability that a drug and a suspected harm are truly related by a non-spurious dose-response curve and offered also a numerical example, based on an association study between paracetamol and asthma. This numerical example agrees with the results already presented in [21].

However, there are some restrictions regarding the presented results that have to be highlighted. First of all, the numerical example tests a limited number of statistical models (see Table 2) and certainly an expansion of this number (like in [22], for instance) would be a welcome extension. Secondly, the example draws on a cohort study, whereas some changes to our model will be required to embed in a full way other kinds of evidence such as those arising from case reports. Moreover, as outlined in Equation (3), in [20,21] the analysis conducted for the probability estimates also included the implementation of modulators, which—for reasons of brevity—have been omitted here. We reserve to rejoinder all these issues in a forthcoming article.

A more general objection regards the use of Bayesian networks per se, in that the underpinning methodology is suspected to rely on “subjective probabilities”. Indeed, unlike in “pure” Bayesian statistics, where one conditionalises on statistical models and hence obtains conditional probabilities mandated by the particular model (parameter), in Bayesian networks one may conditionalise on any proposition (or event), since probabilities are interpreted more widely as one’s uncertainties about general propositions. While a certain degree of subjectivity is undeniable, there is a good argument to be made that some subjectivity is unavoidable in any approach to statistical/uncertain inference [79]. Furthermore a more articulated sense of the Bayesian way to interpret probabilities associated to hypotheses is that of “degree of support” (from the evidence), as opposed to “degree of belief” (see [80]) (What probabilities at a population level may mean to an individual has recently been explored in [81].) In the former sense, the probability does not represent the strength with which an agent may believe in a proposition, but rather, the amount of support provided by a body of evidence with respect to a hypothesis (and its alternatives). Finally, having (un)conditional probabilities being explicitly stated in the network makes the overall inference process more transparent, traceable and ultimately conducive to objectivity, both in the sense expressed by [82], in response to the debate inaugurated among others by Longino’s analysis of the scientific enterprise [83], and in the more traditional sense expressed by Bayesians to this kind of objections, see for instance [84].

## 6. Conclusions

ADRs are responsible for a heavy economic and social burden [15]; additionally, they constitute an extremely vulnerable point for the health system and a key ethical problem for decisions concerning pharmaceutical products. In view of this, the European Parliament and the European Council have changed the regulation of pharmacovigilance practice (Directive 2010/84/EU; Regulation (EU) No 1235/2010, entered into force in July 2012) putting a special emphasis on joint efforts for what can be considered an information-based (rather than power-based) approach to pharmaceutical risk assessment. The related guidelines encourage the integration of information coming from different sources of safety signals (spontaneous case reports, literature, data-mining, pharmacoepidemiological studies, post-marketing trials, drug utilization studies, non-clinical studies, late-breaking information, see also [17]). However, the methodological bases for implementing such a policy are shaky in that causal assessment of ADRs is still parasitic on the (statistical) methods developed to test drug efficacy [85], which are more focused on hypothesis testing, rather than detection and therefore fall short of efficiently using sporadic, fragile and heterogeneous evidence.

Within such perspective, methods for synthesizing heterogeneous evidence are rising as a more and more robust approach to drug safety. While developing its framework on a sound foundational basis, *E-Synthesis* aims to respond to the challenge of merging information from diverse sources by providing a structured way to exploit their joint (dis)confirmatory strength.

The theoretical system developed in [21] is being now translated into a full-fledged computational tool; here we have presented one of its modules: The dose-response line of evidence. Further steps in our research agenda include the development of computational algorithms related to each of the other causality indicators and related evidential modulators. A successful implementation of *E-Synthesis* to better predict ADRs and improve the risk management process (by minimizing both false positives and false negatives) will benefit the pharmaceutical industry, patients and drug agencies.

## Figures and Tables

**Table 1:** Raw data derived from the asthma association study [70] (Table 2). The study lasted from 1990 to 1996 and accounted for 297,282 person-years. In this table *d_i_* represents the dose-level, *n_i_* the number of subjects in each dose-group, *y_i_* the number of subjects with effect in the corresponding dose-group and *r_i_* is the dose-response computed as the ratio *y_i_*/*n_i_*.

*d_i_*	*n_i_*	*y_i_*	*r_i_*
0	137,568	108	7.85 × 10^−4^
10	99,922	112	1.12 × 10^−3^
31.67	32,077	41	1.28 × 10^−3^
60	10,656	16	1.50 × 10^−3^
86.67	17,059	22	1.29 × 10^−3^

**Table 2:** Model evaluation through several criteria: Akaike information criterion (AIC), Bayesian information criterion (BIC), *R*^2^ and adjusted *R*^2^. The best statistical model corresponds to an exponential function where *θ* = (*α*, *β*) = (1.01266 × 10^−3^; 4.17805 × 10^−3^).

Model	*θ*	Specification of *f*(*d_i_*|*θ*)	AIC	BIC	*R* ^2^	Adjusted *R*^2^
exponential	(*α*, *β*)	*α*exp(*βd_i_*)	−66.29	−67.46	0.98	0.97
linear	(*α*, *β*)	*βd_i_* + *α*	−66.79	−67.96	0.56	0.41
quadratic	(*α*, *β*, *γ*)	*γd_i_*^2^ + *βd_i_* + *α*	−74.57	−76.13	0.95	0.90
cubic	(*α*, *β*, *γ*, *δ*)	*δd_i_*^3^ + *γd_i_*^2^ + *βd_i_* + *α*	−70.10	−72.06	0.95	0.80

**Table 3:** Predicted values through exponential model.

${\hat{r}}_{i}$
1.01 × 10^−3^
1.06 × 10^−3^
1.16 × 10^−3^
1.30 × 10^−3^
1.46 × 10^−3^

## Abbreviations

The following abbreviations are used in this manuscript:

*A*	Adjustment for Confounders
ADR	Adverse drug reaction
AIC	Akaike information criterion
*B*	Blinding
BIC	Bayesian information criterion
*D*	Duration
*DR*	Dose-response Relationship
ECETOC	European Centre for Ecotoxicology and Toxicology of Chemicals
*ES*	Effect Size
EUDRAVIGILANCE	European Union Drug Regulating Authorities Pharmacovigilance
FAERS	FDA Adverse Event Reporting System
FDA	Food and Drug Administration (US)
IC	Information Component
*M*	Mechanistic Knowledge
MCP-Mod	Multiple Comparison Procedures and Modeling algorithm
MGPS	Multi-item Gamma Poisson Shrinker
*PD*	Probabilistic Dependence
PRR	Proportional Reporting Ratio
*R*	Randomisation
RCT	Randomized controlled trial
*REP*	Report Variable
*RoG*	Rate of Growth
ROR	Reporting Odds Ratio
*SB*	Sponsorship Bias
*SS*	Sample Size
*T*	Temporal Precedence
WHO	World Health Organization
*©*	Hypothesis of Causation
Δ	Difference Making
E	Available Evidence
Σ	Set of statistical Indicators: *PD*, *DR* and *RoG*
